# Development and Evaluation of a GPT4-Based Orofacial Pain Clinical Decision Support System

**DOI:** 10.3390/diagnostics14242835

**Published:** 2024-12-17

**Authors:** Charlotte Vueghs, Hamid Shakeri, Tara Renton, Frederic Van der Cruyssen

**Affiliations:** 1 Department of Oral and Maxillofacial Surgery, University Hospitals Leuven, 3000 Leuven, Belgium; 2 Department of Oral Surgery, King’s College London Dental Institute, London SE5 9RW, UK; 3 OMFS-IMPATH Research Group, KU Leuven, 3000 Leuven, Belgium

**Keywords:** validation, development, large language model, GPT4, clinical decision support system

## Abstract

**Background**: Orofacial pain (OFP) encompasses a complex array of conditions affecting the face, mouth, and jaws, often leading to significant diagnostic challenges and high rates of misdiagnosis. Artificial intelligence, particularly large language models like GPT4 (OpenAI, San Francisco, CA, USA), offers potential as a diagnostic aid in healthcare settings. **Objective**: To evaluate the diagnostic accuracy of GPT4 in OFP cases as a clinical decision support system (CDSS) and compare its performance against treating clinicians, expert evaluators, medical students, and general practitioners. **Methods**: A total of 100 anonymized patient case descriptions involving diverse OFP conditions were collected. GPT4 was prompted to generate primary and differential diagnoses for each case using the International Classification of Orofacial Pain (ICOP) criteria. Diagnoses were compared to gold-standard diagnoses established by treating clinicians, and a scoring system was used to assess accuracy at three hierarchical ICOP levels. A subset of 24 cases was also evaluated by two clinical experts, two final-year medical students, and two general practitioners for comparative analysis. Diagnostic performance and interrater reliability were calculated. **Results**: GPT4 achieved the highest accuracy level (ICOP level 3) in 38% of cases, with an overall diagnostic performance score of 157 out of 300 points (52%). The model provided accurate differential diagnoses in 80% of cases (400 out of 500 points). In the subset of 24 cases, the model’s performance was comparable to non-expert human evaluators but was surpassed by clinical experts, who correctly diagnosed 54% of cases at level 3. GPT4 demonstrated high accuracy in specific categories, correctly diagnosing 81% of trigeminal neuralgia cases at level 3. Interrater reliability between GPT4 and human evaluators was low (κ = 0.219, *p* < 0.001), indicating variability in diagnostic agreement. **Conclusions:** GPT4 shows promise as a CDSS for OFP by improving diagnostic accuracy and offering structured differential diagnoses. While not yet outperforming expert clinicians, GPT4 can augment diagnostic workflows, particularly in primary care or educational settings. Effective integration into clinical practice requires adherence to rigorous guidelines, thorough validation, and ongoing professional oversight to ensure patient safety and diagnostic reliability.

## 1. Introduction

Pain is the most common reason to seek care [1]. The Institute of Medicine and the American Pain Society report that 100 million adults are affected by pain, costing $635 billion each year in treatment and lost productivity, which is likely to increase with the world’s aging population [2]. Approximately 40% of the global burden of disease is attributable to trigeminal pain (including toothache, headaches, temporomandibular disorders, and more than 160 other orofacial pain conditions) [3,4]. Appropriate diagnosis and treatment of chronic pain can substantially lower morbidity, mortality, and healthcare costs [5].

Misdiagnosis of chronic pain is common, with 5% of adult US patients experiencing diagnostic errors [6]. Researchers from Johns Hopkins Hospital reported that 40–80% of chronic pain patients are misdiagnosed [7]. In addition, diagnostic errors resulted in over 25% of claims and 35% of malpractice payments in the US [8]. The high prevalence of misdiagnosis stems from several factors [7,9,10]. Chronic pain conditions, particularly orofacial pain (OFP), often involve complex, overlapping symptoms that mimic other disorders, leading to diagnostic uncertainty. Additionally, a lack of knowledge and training and the subjective nature of pain assessment exacerbate these challenges, often resulting in delayed or inappropriate treatments, patient harm, and increased healthcare burdens.

Orofacial pain refers to any pain localized to the region of the face, mouth, or jaws, often associated with conditions affecting nerves, muscles, or other related structures. It encompasses a wide range of disorders, each with distinct clinical features. Accurate diagnosis requires a comprehensive understanding of the International Classification of Orofacial Pain (ICOP) and International Classification of Headache Disorders frameworks, which categorize these conditions systematically [11,12]. ICOP, developed by a group of international orofacial pain experts, provides a structured approach to distinguishing orofacial pain disorders, facilitating more accurate diagnosis and treatment. Advancements in artificial intelligence, particularly in large language models like GPT4 (OpenAI, San Francisco, CA, USA), have opened new possibilities across various sectors, including healthcare [13,14]. ChatGPT, powered by the advanced GPT4 language model, is designed to interpret complex natural language inputs, enabling it to generate sophisticated, human-like responses [15]. This potential has led to increasing interest in exploring its role in assisting medical diagnostics, including conditions like orofacial pain, which often require nuanced differential diagnosis due to their complexity [16,17].

The diagnosis of OFP is inherently complex due to the vast number of structures supplied by the trigeminal nerve in the head, face, and mouth, which presents an immediate diagnostic challenge [18]. Toothache, the most common pain condition globally, can be refractory and mimic various headache and other OFP conditions, adding further difficulty [19]. The presence of siloed clinical specialties, each operating within their own domains (e.g., dentists focusing on dental treatments, ENT surgeons correcting sinus issues, and neurologists providing medication), can lead to ill-informed practices, delayed diagnoses, and subsequent patient harm [20,21]. Delays in diagnosis are not uncommon, with studies showing an average time of nine months from onset of pain to seeking medical attention and an additional eight months to reach a correct cancer diagnosis, often mistaking OFP for odontogenic or neuropathic causes [22,23,24]. Diagnostic delays are higher in idiopathic facial pain, with an average delay of 19.3 months, and patients often consult multiple clinicians, leading to unnecessary interventions [22]. The economic and emotional costs are significant, as illustrated in a Korean study where diagnostic delays averaged 34.8 months, often resulting in invasive dental treatments and substantial financial burdens [9].

This complexity is compounded by misdiagnosis of primary headaches, such as migraine, which is underrecognized in OFP patients despite being a common global health burden [25]. An increasing number of primary headaches are also being diagnosed within multidisciplinary OFP clinics, often misclassified initially as trigeminal neuralgia or other conditions [26]. Poorly managed or misdiagnosed pain remains a significant source of patient complaints and adverse events, highlighting the importance of both accurate diagnosis and effective patient communication to manage expectations [27,28].

Diagnosing orofacial pain remains a challenge, even for experienced clinicians, highlighting the potential value of AI tools as diagnostic aids [29]. Tools such as GPT4 present a promising solution to these issues by offering enhanced diagnostic accuracy and structured differential diagnoses. Leveraging AI’s ability to process large volumes of complex clinical data, these tools can identify nuanced patterns and correlations that might elude human evaluators. AI’s systematic and unbiased approach can help standardize diagnoses, improve adherence to frameworks such as ICOP, and assist clinicians in narrowing down potential conditions more efficiently. By addressing the diagnostic variability inherent in chronic pain cases, AI has the potential to reduce misdiagnoses, shorten diagnostic delays, and ultimately improve patient outcomes.

In this study, we evaluate the diagnostic accuracy of GPT4 in cases of orofacial pain as a clinical decision support system (CDSS). Our objective is twofold: to validate GPT4’s performance and to compare it against treating clinicians, human experts, and medical students. By systematically comparing these diagnostic methods, this research aims to assess whether GPT4 can reliably serve as a supplementary tool in clinical practice to enhance diagnostic precision and ultimately improve patient care.

## 2. Materials and Methods

### 2.1. Study Design

This study aimed to validate the diagnostic accuracy of GPT4 in orofacial pain cases and compare its performance against treating clinicians, expert evaluators, medical students, and general practitioners. This study was conducted using anonymized case descriptions obtained from existing clinical records obtained from the Department of Oral and Maxillofacial Surgery, University Hospitals Leuven, and the orofacial pain clinic within this department between 2020 and 2024.

### 2.2. Data Collection

The dataset consisted of 100 anonymized patient case descriptions involving different orofacial pain conditions. These anonymized cases were collected from University Hospitals Leuven. Each case was thoroughly vetted to ensure diverse representation of orofacial pain conditions, including dental pain, myofascial pain, temporomandibular disorders, trigeminal neuralgia, and idiopathic facial pain syndromes.

### 2.3. Prompt Design

To ensure a comprehensive evaluation of each case, we developed a structured prompt (Supplemental Table S1), incorporating all available clinical information. The prompt was designed to systematically elicit key diagnostic features, enabling GPT4 to generate both a primary diagnosis and a differential diagnosis using either the International Classification of Orofacial Pain (ICOP) or the International Classification of Headache Disorders, 3rd edition (ICHD-3) criteria [11,12]. By using these standardized diagnostic frameworks, the model’s outputs were directly comparable to established clinical standards, enhancing the reliability of the assessment. The prompt included only textual information, such as patient history, symptom characteristics, clinical findings, and relevant diagnostic test results, and utilized validated clinical scales to provide clarity in interpreting key scores. This structured approach ensured that the model’s diagnostic decisions were specific, consistent with recognized criteria, and supported a robust differential diagnosis ranked by probability, thereby offering a thorough evaluation of each orofacial and headache pain case.

### 2.4. Validation Process

GPT4 was used to provide a diagnosis for each of the 100 orofacial pain cases. Each case description was independently reviewed, and the model generated a primary diagnosis along with a differential diagnosis according to ICOP. The outcomes provided by GPT4 were compared with the gold-standard diagnoses that were established by the treating clinicians from the medical records.

The ICOP system was used to assess diagnostic accuracy. ICOP categorizes orofacial pain into different levels, providing a structured and hierarchical framework for classification. ICOP structures the first level into six main categories, referred to as first-digit levels:

1. Orofacial pain attributed to disorders of dentoalveolar and anatomically related structures;
2. Myofascial orofacial pain;
3. Temporomandibular joint (TMJ) pain;
4. Orofacial pain attributed to lesion or disease of the cranial nerves;
5. Orofacial pains resembling presentations of primary headaches;
6. Idiopathic orofacial pain.

Level 2 provides a more detailed categorization, specifying subtypes within the broader categories, such as distinguishing between primary and secondary myofascial pain. Level 3 offers the most specific diagnosis, identifying the exact condition (Table 1).

In this study, the accuracy was assessed based on how well the model’s diagnosis matched the correct ICOP level. A correct diagnosis at level 3 was considered the highest level of accuracy, indicating that the model correctly identified the specific condition. A level 2 diagnosis was considered moderately accurate, indicating that the model correctly identified the subtype but not the specific condition. A level 1 diagnosis was considered low accuracy, as it only matched the general category of orofacial pain. Cases where the model provided an incorrect diagnosis were classified as wrong diagnoses. This produced the following scoring system, which was used to evaluate the primary diagnosis.

- 0: Wrong diagnosis;
- 1: Correct diagnosis at level 1;
- 2: Correct diagnosis at level 2;
- 3: Correct diagnosis at level 3.

This diagnostic performance scoring method allowed for a maximum possible score of 300 (100 cases × 3 points per case).

To evaluate the relevance of the differential diagnoses provided by the model, we adopted a similar method published by Kanjee et al. [30]. For each case, the model was tasked with producing a list of possible diagnoses, ranked by likelihood. Next, we scored each differential list using the following differential quality scoring system:

- 0: No suggestions close to the target diagnosis;
- 1: The suggestions included something related but unlikely to be helpful;
- 2: The suggestions included something closely related that might have been helpful;
- 3: The suggestions included something very close but not exact;
- 4: The actual diagnosis was suggested in the differential;
- 5: Correct final diagnosis by GPT4.

This scoring method allowed for a maximum possible score of 500 (100 cases × 5 points per case).

### 2.5. Comparative Analysis

In addition, a subset of 24 cases was selected to perform a comparative analysis. These 24 cases were assessed by four different groups of assessors: (1) GPT4, (2) two clinical experts with significant experience in orofacial pain, (3) two final-year medical students, and (4) two general practitioners. Each participant was tasked with diagnosing each case up to the third-digit level. Their diagnoses were then compared to the clinicians’ original diagnoses, which served as the gold standard. The assessors were blinded from each other and the clinician’s original diagnosis, and they were not aware their results would be compared to GPT4. The accuracy outcomes from all evaluators were assessed using the diagnostic performance score described above. This scoring method allowed for a maximum possible score of 72 for each evaluator (24 cases × 3 points per case). The cumulative scores provided a measure of each participant’s diagnostic performance across all cases, enabling a comparative analysis between the different groups.

### 2.6. Interrater Reliability

To assess consistency between evaluators, Fleiss’ multirater kappa analysis was conducted. This analysis measured interrater agreement across the four groups and GPT4. The goal was to determine the level of agreement between GPT4’s diagnostic decisions and those made by various healthcare professionals. A kappa value was computed to establish the overall level of concordance.

### 2.7. Outcome Measures

The primary outcome measure was the diagnostic performance of GPT4 in correctly diagnosing orofacial pain cases to the highest ICOP level in comparison to the reference diagnosis made by the treating physician (golden standard) and different groups of assessors. Secondary outcome measures included the ability to provide accurate differential diagnoses and the interrater reliability scores compared to other evaluators.

### 2.8. Statistical Analysis

Statistical analyses were conducted using SPSS (version 27.0, Armonk, NY, USA). Descriptive statistics were used where appropriate. Fisher’s exact test was used for evaluating significant differences between diagnostic groups, with a significance threshold of *p* < 0.05. Fleiss’ multirater kappa values were interpreted based on standard benchmarks to understand interrater agreement.

## 3. Results

### 3.1. Study Sample Characteristics

This study evaluated a total of 100 cases of orofacial pain for diagnostic validation of GPT4. The distribution of diagnoses in this study included various categories, such as dental pain (5%), primary and secondary myofascial pain (13%), temporomandibular joint disorders (11%), neuropathic pain, including trigeminal neuralgia (36%), and idiopathic facial pain syndromes (15%). The sample included 44 males and 56 females, with an average age of 48 years, ranging from 16 to 86 years (Table 2).

### 3.2. Diagnostic Performance of GPT4

**Figure 1:**
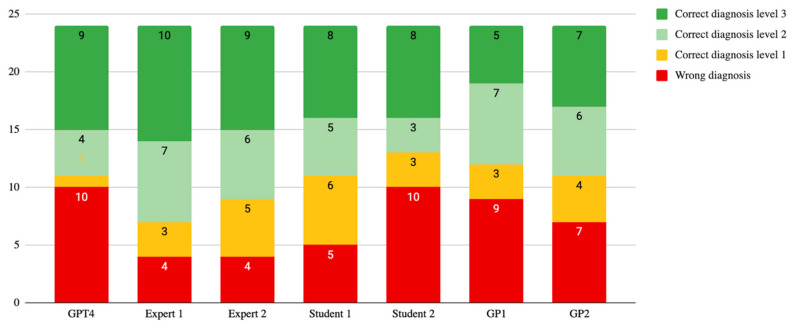
Comparison of diagnostic performance of GPT4 and different assessors: orofacial pain experts, students, and general practitioners (GPs).

GPT4 demonstrated varying levels of diagnostic performance across the 100 evaluated cases. The model achieved the highest accuracy level (level 3) in 38% of cases, correctly identifying the primary diagnosis. Moderate accuracy (level 2) was observed in 16% of cases, while low accuracy (level 1) was observed in 11% of cases (Figure 1). In 35% of cases, GPT4 provided an incorrect primary diagnosis.

**Figure 2:**
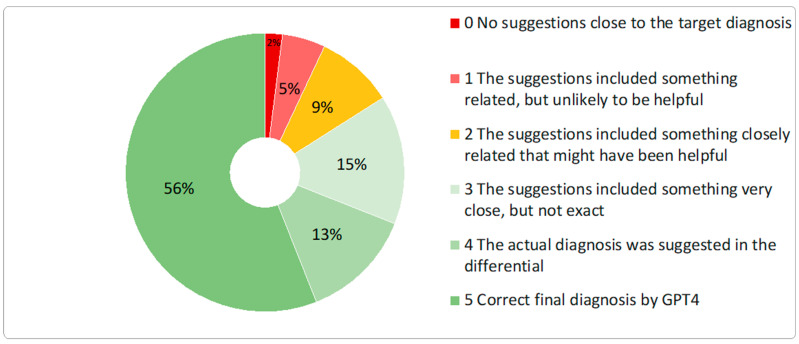
Differential quality score of the GPT4 model.

The overall diagnostic performance score for GPT4 was 157 out of a possible 300 points (52%). The differential quality score, reflecting the accuracy of differential diagnoses, was 400 out of 500 points (80%) (Figure 2).

### 3.3. Comparative Analysis of Evaluators

For the subset of 24 cases evaluated by multiple assessors, the model’s performance was compared with that of clinical experts, medical students, and general practitioners. GPT4 correctly identified the diagnosis at the highest level (level 3) in 9 out of 24 cases (37.5%). Clinical experts outperformed GPT4, correctly diagnosing 13 out of 24 cases (54%). GPT4 achieved a diagnostic performance score of 36 out of a maximum of 72, while Expert 1 and Expert 2 outperformed GPT4 with scores of 47 and 44, respectively (Table 3).

Fisher’s exact test was used to assess the statistical significance of the differences in diagnostic performance between GPT4 and the other assessors. The *p*-values for most comparisons did not show statistically significant differences (*p* > 0.05), except for GP 1 and GP 2, where the *p*-values were 0.030 and 0.046 (Table 4), respectively, indicating significant differences in diagnostic performance compared to GPT4.

### 3.4. Interrater Agreement

The interrater agreement between GPT4 and the human evaluators was assessed using Fleiss’ multirater kappa analysis (Table 5). The overall kappa value for agreement between all evaluators was 0.219 (*p* < 0.001), indicating low agreement. The highest level of agreement was observed among general practitioners (kappa = 0.488, *p* < 0.001). Agreement between GPT4 and clinical experts was lower (kappa = 0.145, *p* = 0.042), suggesting limited agreement between the model and expert-level evaluations.

### 3.5. Performance in Specific Diagnostic Categories

The model performed well in dentoalveolar-related pain and cranial nerve-related pain diagnoses, achieving 79% and 71% accuracy, respectively (Table 6). Its accuracy was moderate for orofacial pain resembling primary headaches (63%) but lower for myofascial orofacial pain (13%), temporomandibular joint pain (30%), and idiopathic orofacial pain (31%).

Table 7 further stratifies GPT4’s performance by comparing it to human evaluators (experts, students, and GPs) across ICOP level 1 categories. The model matched human evaluators well in dentoalveolar-related pain (78% accuracy) and cranial nerve-related pain (48%, near the 51% human mean). For myofascial orofacial pain (33%) and idiopathic orofacial pain (22%), GPT4 scored below the human mean (43%). However, for orofacial pain resembling primary headaches, it outperformed human evaluators, achieving 50% accuracy compared to the human mean of 38%.

GPT4 also showed stronger diagnostic accuracy for certain conditions, such as trigeminal neuralgia (Table 8). Among the 16 cases involving trigeminal neuralgia, GPT4 correctly diagnosed 81% of cases at the highest level of accuracy (level 3). The model provided incorrect diagnoses in only 13% of these cases. The overall quality score for trigeminal neuralgia cases was 41 out of 48 points (85%), and the differential quality score was 74 out of 80 points (93%).

### 3.6. Implementation

The diagnostic model described is integrated into a free-to-use website, https://FaceYourPain.org (TrigeminalNerve Ltd., London, UK), which is currently in beta testing. This platform allows users to input case details related to orofacial and headache pain and receive a diagnostic assessment based on the model’s interpretation of the provided information. By offering this tool in an easy, accessible format, FaceYourPain.org aims to provide a valuable resource for healthcare professionals and individuals seeking guidance on these challenging pain conditions during the beta phase while gathering feedback to refine and improve the system’s functionality.

## 4. Discussion

Our evaluation of GPT4 as a clinical decision support system (CDSS) for orofacial pain demonstrates significant potential in handling complex and nuanced diagnostic cases. GPT4 achieved an overall diagnostic performance score of 52%, ranging from 13% to 79% when stratified for different diagnostic groups. The highest level of accuracy (level 3) was obtained in 38% of cases. It provided an incorrect primary diagnosis in 35%, which was in the same range as the non-expert evaluators. Notably, the model exhibited stronger diagnostic accuracy for certain conditions, such as dentoalveolar pain conditions and trigeminal neuralgia. Among the 16 cases involving trigeminal neuralgia, GPT4 correctly diagnosed 81% at the highest level of accuracy (level 3) and provided incorrect diagnoses in 13% of these cases. The overall quality score for trigeminal neuralgia cases was 41 out of 48 points (85%), and the differential diagnosis quality score was 74 out of 80 points (93%). The model’s performance was comparable to non-expert human evaluators but could not yet exceed orofacial pain experts.

This specificity is promising for the targeted use of AI in the differential diagnosis of certain conditions. It also highlights the model’s lower diagnostic accuracy for other conditions and thus the necessity for further training with more diverse and specialized clinical datasets. These findings align with those of Kanjee et al., who assessed GPT4’s accuracy in resolving challenging diagnostic dilemmas from *New England Journal of Medicine* clinicopathologic conferences [30]. In their study, GPT4 provided the correct diagnosis in 64% of cases, underscoring its capability in complex clinical reasoning contexts.

Misdiagnosis and delayed diagnosis in patients with chronic orofacial pain (OFP) conditions are frequently reported, often leading to significant patient harm. Studies indicate that patients typically consult multiple physicians and receive various treatments before obtaining an accurate diagnosis, with reported delays ranging from 12 to 20 months or, in some cases, up to 34.8 months [9]. Delays tend to be longer for patients with intraoral pain compared to those with extraoral pain, and patients endure an average of 3.7 consultations and 2.3 misdiagnoses before a correct diagnosis is determined. Notably, 73.3% of patients underwent unnecessary surgical or dental interventions. In a cohort of 500 patients with burning mouth syndrome, an average of three specialists were consulted, with a mean number of over three misdiagnoses per patient [10].

Primary headaches are also significantly underdiagnosed in OFP patients, despite a global migraine prevalence of 14–15%, accounting for 4.9% of global ill health in terms of years lived with disability [25]. Migraine often presents as facial pain but is not commonly recognized by clinicians. Neurovascular conditions, linked to primary headaches, are now formally recognized in the International Classification of Orofacial Pain (ICOP) [12]. Patients with chronic OFP often seek care across various healthcare domains, including general dental practitioners, who are not well equipped, educated, or funded to manage OFP cases [26]. The recent crisis in access to dental care has exacerbated this issue, with more patients turning to medical settings, emergency rooms, and oral and maxillofacial surgery services for treatment [31].

To improve the care of OFP patients, several strategies have been proposed, such as increasing access to multidisciplinary clinics and incorporating pre-screening procedures. Psychosocial pre-screening and brief psychological assessments in primary care are recommended to enhance treatment outcomes and guide tailored treatment decisions [26]. Recent studies also suggest the potential of self-screening tools for patients, including validated self-report instruments that assess musculoskeletal, neuropathic, or neurovascular origins of pain [32]. Moreover, precision medicine approaches are being explored to improve OFP care, though none have developed an online tool for a comprehensive, ICOP-aligned assessment—a gap our model aims to address [33,34].

Compared to the current state of diagnosis for orofacial pain (OFP), which is marked by high misdiagnosis rates and significant delays, the application of GPT4 shows promise in improving diagnostic accuracy, efficiency, and patient outcomes. The integration of GPT4 into diagnostic pathways could mitigate these issues by enhancing the speed and accuracy of diagnosis, providing faster signposting to appropriate specialists, and reducing patient exposure to ineffective or invasive treatments. By utilizing a tool trained to recognize patterns across a wide range of symptoms and conditions, our proposed model could facilitate a more precise identification of conditions such as burning mouth syndrome and trigeminal autonomic cephalalgias—conditions that currently take between 5.6 and 8.7 years to diagnose but could potentially be identified within just 15 min using our tool. This approach not only lessens patient distress and the burden on healthcare resources but also offers a pathway to a more efficient, data-supported diagnostic process, ultimately aiming to reduce the economic and physical toll of protracted OFP diagnostic journeys.

The model’s strength in generating meaningful differential diagnoses in 80% of cases, even in challenging scenarios like rare conditions or presentations with limited prior data, is particularly noteworthy. This is especially relevant in pain medicine, where conditions like atypical odontalgia can be misclassified due to symptom overlap with other dental or neurological issues [35]. Including high-quality differential diagnoses, as demonstrated in our study, assists clinicians by presenting a range of potential conditions to consider, thereby reducing oversight and facilitating earlier, more accurate management. Additionally, we believe these tools have potential in addressing patient satisfaction and compliance, which are known to be low in chronic pain patients [36]. GPT4 could help address these challenges by improving diagnostic accuracy and fostering clear communication. Its ability to provide structured, transparent differential diagnoses enhances patient understanding and trust in care. By streamlining the diagnostic process and reducing unnecessary interventions, GPT4 can improve patient satisfaction and adherence to treatment plans. Additionally, patient-facing tools powered by GPT4 could empower individuals with early and continued guidance, fostering a sense of control and engagement in their healthcare journey.

GPT4’s ability to integrate structured prompts and apply the International Classification of Orofacial Pain (ICOP) illustrates how AI can effectively handle structured and semi-structured data in combination with diagnostic frameworks, contributing to a more systematic diagnostic approach. This aligns with findings from Benoliel et al., who highlighted the complexities of accurately diagnosing orofacial pain using existing classification systems that are not always comprehensive or integrated [29]. By considering a broad spectrum of possible conditions, GPT4 can help mitigate some of these diagnostic gaps and improve clinical implementation of diagnostic classification and coding frameworks [37].

The challenge of diagnosing orofacial pain is underscored by the fair to moderate interrater reliability scores in this study. This suggests that while GPT4 may identify key features of orofacial pain, its decision-making pathways do not consistently align with those of humans. Another reason for the low interrater agreement might be that this study was underpowered. The relatively small sample size and the limited number of cases for each condition could contribute to variability in agreement levels. Conducting a larger-scale study with a sufficient number of labeled case notes for each condition could help resolve this issue and provide a more accurate assessment of interrater reliability and diagnostic accuracy.

While AI’s role in synthesizing multimodal data to improve diagnostic accuracy has been emphasized and questioned by some, our study focused on textual data, primarily clinical history [13,38,39]. Integrating multimodal data—including imaging and other diagnostic and patient-reported inputs—and involving a wider spectrum of orofacial pain cases with varied demographics could enhance GPT4’s diagnostic accuracy and provide a more complete understanding of the patient’s condition. Another way to enhance its performance is by leveraging clinician and specialist feedback during the refinement process, enabling the model to better align with expert diagnostic reasoning. Optimizing structured prompt design to systematically elicit relevant diagnostic details could improve the model’s consistency and specificity. The natural evolution of AI models could further bring improved accuracy, as evidenced by the release of two new OpenAI models (4o and o1) during the writing of this paper. To effectively integrate GPT4 into clinical workflows, a hybrid framework that combines AI-driven analytics with human clinical oversight is essential [40,41,42]. This approach maximizes accuracy while maintaining safety and ethical standards. Rigorous real-world validation in clinical settings, coupled with the establishment of clear operational guidelines, is crucial for ensuring reliable implementation. By leveraging AI’s computational ability to process multiple variables simultaneously and clinicians’ expertise in interpreting patient-specific nuances, such frameworks can enhance diagnostic accuracy. This is particularly beneficial for conditions characterized by diagnostic uncertainty, such as complex orofacial pain syndromes. Finally, rigorous real-world validation in clinical settings and the establishment of clear operational guidelines are crucial for integrating these tools effectively into diagnostic workflows, thereby reducing variability and enhancing patient care.

A strength of this study is its systematic evaluation of GPT4 using the ICOP framework, ensuring that results are based on recognized diagnostic criteria and facilitating robust comparisons across different evaluators. However, several limitations need to be considered. The dataset of 100 cases, although diverse, may not encompass all possible presentations of orofacial pain, potentially limiting the generalizability of the findings. GPT4’s reliance on text-based inputs means that important diagnostic clues obtained through physical examination or patient interaction may be missed. We recognize the absence of a feature-importance or sensitivity analysis to evaluate the impact of individual diagnostic features or levels on GPT4’s performance. While such an approach could provide insights into the specific contributions of various features, it was not part of our study design. We believe this would oversimplify real-world clinical scenarios, where clinicians typically rely on comprehensive information to make diagnostic decisions. Artificially removing features (e.g., column dropping) may not reflect the integrated and nuanced nature of clinical workflows, thereby limiting the applicability of such an analysis to practical settings. The findings of our study revealed the inherent convolution of diagnosing orofacial pain, particularly at deeper ICOP levels. Despite using the treating physician’s diagnosis as the gold standard, it must be acknowledged that even these diagnoses are not infallible. The subjective nature of clinical assessments and their inherent variability, compounded by the nuanced presentation of orofacial pain disorders, implies that the “gold standard” may itself harbor inaccuracies leading to over- or underdiagnosis. This is further reinforced by the fact that the experts failed to achieve a 100% diagnostic accuracy and low interrater agreement scores, indicating substantial variability in diagnosing these cases.

The findings from this study underscore the potential of AI, specifically GPT4, as a supportive tool in the diagnostic process for orofacial pain. While GPT4 is not yet ready to replace human clinicians, it can effectively augment diagnostic workflows, particularly in primary care or training environments. The model’s demonstrated accuracy in conditions like trigeminal neuralgia suggests potential specialized applications that could be explored further. In settings where access to specialized expertise is limited, GPT4’s high-quality differential diagnoses can aid in narrowing down potential conditions, supporting timely and effective referrals. While these advancements hold the potential to streamline diagnostic workflows and free up valuable clinician time, the assumption that this time will automatically address workforce gaps or lead to increased productivity is overly simplistic. Instead, studies indicate that freed-up time may often be redirected toward other essential but non-clinical responsibilities, such as training, management, or administrative tasks [43]. This underscores the need for thoughtful integration strategies that acknowledge AI’s complementary role in augmenting human decision-making rather than replacing it.

The cost implications cannot be overlooked. Implementation demands substantial financial investment in infrastructure, staff training, and continuous model validation [44,45,46]. The development and enforcement of rigorous standards and guidelines to safeguard patient safety and diagnostic reliability further add to the financial burden. These expenses might encompass hiring specialized technical staff, acquiring high-performance computing resources, and maintaining iterative testing and monitoring frameworks. While these investments may yield long-term efficiencies by streamlining workflows and enhancing care delivery, the financial viability of such initiatives hinges on a careful assessment of short-term costs versus anticipated benefits.

For now, however, it remains challenging to accurately estimate the financial impact of deploying these tools, as costs will vary depending on factors such as system scale, the complexity of integration, and the pace of technological advancements. This uncertainty underscores the need for adaptive financial planning and ongoing evaluation to ensure that the benefits of AI adoption justify the associated expenses.

The need for rigorous standards and guidelines in AI-driven healthcare systems is crucial to ensure patient safety and diagnostic reliability [47]. Implementing such guidelines in deploying GPT4 as a CDSS will address ethical concerns and ensure responsible use in clinical environments. These safeguards are essential given that AI models, including GPT4, often function as “black boxes”, where the reasoning behind specific outputs may not always be transparent [30]. To further address the challenge of validating and implementing the latest AI models, which are released at a pace that outstrips the current scientific and regulatory framework, healthcare systems must adopt adaptive validation processes. This includes developing iterative testing protocols that allow for continuous monitoring and assessment of AI models in real-world clinical settings. Collaboration between regulatory bodies, academic researchers, and industry stakeholders is essential to establish agile yet robust standards that balance innovation with safety and efficacy. Such mechanisms should prioritize transparency, reproducibility, and regular updates to guidelines, ensuring that the rapid evolution of AI aligns with the ethical and clinical demands of modern healthcare.

The recently introduced EU AI Act presents significant legal and ethical implications addressing these concerns [48]. Legally, the categorization implicates GPT4 under stringent compliance measures intended for high-risk AI systems, including mandates for transparency, risk management, and data governance. This classification recognizes the potential of such AI to propagate misinformation, exacerbate biases, and influence decision-making in critical areas like healthcare. Ethically, it raises questions about the balance between leveraging AI for innovation and ensuring its responsible use to prevent harm. For example, when employed as a clinical decision support tool, like in the diagnosis of orofacial pain, GPT4’s reliance on training data and algorithmic processes may unintentionally perpetuate diagnostic inaccuracies or bias, disproportionately affecting marginalized groups. Adhering to the EU AI Act, among others, requires addressing these risks while preserving patient trust and safety through robust validation, ongoing oversight, and ethical safeguards.

## 5. Conclusions

GPT4 shows substantial promise as a CDSS for orofacial pain by improving diagnostic accuracy, offering structured differential diagnoses, and potentially reducing unnecessary interventions. Effective integration of AI tools like GPT4 within clinical workflows will require adherence to high-quality guidelines, thorough validation, and ongoing oversight by healthcare professionals to ensure patient safety and diagnostic reliability. Future research should focus on evaluating GPT4’s performance in real-world clinical settings, understanding its diagnostic limitations, and continuously refining its algorithms to better serve both patients and clinicians.

## Figures and Tables

**Table 1:** International Classification of Orofacial Pain (ICOP) hierarchical classification system. Example of the hierarchical classification of myofascial orofacial pain (level 1) into two deeper levels to reach a final diagnosis.

Level	Description
1	2. Myofascial orofacial pain
2	2.1 Primary myofascial orofacial pain
3	2.1.1 Acute primary myofascial orofacial pain
3	2.1.2 Chronic primary myofascial orofacial pain
2	2.2 Secondary myofascial orofacial pain
3	2.2.1 Myofascial orofacial pain attributed to tendonitis
3	2.2.2 Myofascial orofacial pain attributed to myositis
3	2.2.3 Myofascial orofacial pain attributed to muscle spasm

**Table 2:** Study sample characteristics.

	GPT4 Evaluation *n* = 100	Comparison Agreement *n* = 24
Male/female ratio	44/56 (0.79)	11/13 (0.85)
Age, years		
Mean	48 y	51 y
Range	16 y–86 y	28 y–86 y
1.1 Dental pain	5	3
1.2 Oral mucosal, salivary gland, and jawbone pains	3	3
2.1 Primary myofascial orofacial pain	5	1
2.2 Secondary myofascial orofacial pain	8	2
3.1 Primary temporomandibular joint pain	5	0
3.2 Secondary temporomandibular joint pain	6	1
4.1 Pain attributed to lesion or disease of the trigeminal nerve	31	7
4.2 Pain attributed to lesion or disease of the glossopharyngeal nerve	5	0
5.1 Orofacial migraine	4	1
5.2 Tension-type orofacial pain	2	1
5.3 Trigeminal autonomic orofacial pain	8	2
5.4 Neurovascular orofacial pain	3	0
6.1 Burning mouth syndrome (BMS)	6	1
6.2 Persistent idiopathic facial pain (PIFP)	4	1
6.3 Persistent idiopathic dentoalveolar pain	3	0
6.4 Constant unilateral facial pain with additional attacks (CUFPA)	2	1

**Table 3:** Comparison of diagnostic performance between GPT4 and different assessors: orofacial pain experts, students, and general practitioners (GPs).

	GPT4	Expert 1	Expert 2	Student 1	Student 2	GP 1	GP 2
Total diagnostic performance score (range 0–72)	36 (50%)	47 (65%)	44 (61%)	39 (56%)	33 (46%)	32 (44%)	37 (51%)

**Table 4:** Pairwise comparisons of performance between GPT4 and the other answer providers.

Answer Provider	Diagnostic Performance Score (Max = 72)	Fisher’s Exact Value	*p*-Value
GPT4	36	Reference	
Expert 1	47	7.958	0.646
Expert 2	44	9.474	0.356
Student 1	39	10.123	0.295
Student 2	33	11.948	0.126
GP 1	32	14.536	0.030
GP 2	37	13.751	0.046

**Table 5:** Overall interrater agreement (Fleiss’ multirater kappa analysis).

Raters	Kappa	*p*-Value	95.0% CI for Kappa	
			Lower	Upper
All	0.219	<0.001	0.174	0.264
GPT4/experts	0.145	0.042	0.005	0.285
Students/experts	0.163	<0.001	0.067	0.260
GP/experts	0.235	<0.001	0.139	0.332
GP/students	0.310	<0.001	0.214	0.407
GP/GPT4	0.360	<0.001	0.219	0.501
Students/GPT4	0.278	<0.001	0.137	0.419
GP/GP	0.488	<0.001	0.250	0.725
Student/student	0.313	0.010	0.075	0.552
Expert/expert	0.183	0.135	−0.057	0.424

**Table 6:** Diagnostic performance of the GPT4 model on the first ICOP level for 100 case vignettes. Score is given in absolute and relative (%) numbers. The diagnostic performance was scored on a scale from 0 to 3 points for each case, with higher scores reflecting greater diagnostic precision.

ICOP Level 1	Number of Cases	Max Possible Score	GPT Diagnostic Performance	
1 Dentoalveolar-related pain	8	24	19	79%
2 Myofascial orofacial pain	13	39	5	13%
3 Temporomandibular joint pain	11	33	10	30%
4 Pain related to lesions or disease of the cranial nerves	36	108	77	71%
5 Orofacial pain resembling primary headaches	17	51	32	63%
6 Idiopathic orofacial pain	15	45	14	31%
Total	100	300	157	52%

**Table 7:** Diagnostic performance of the GPT4 model stratified according to the International Classification Orofacial Pain level 1. GP: general practitioner. The diagnostic performance was scored on a scale from 0 to 3 points for each case, with higher scores reflecting greater diagnostic precision. Each evaluator’s total score is given in absolute and relative (%) figures. The mean human evaluator score is the mathematical mean of experts 1–2, students 1–2 and GPs 1–2.

ICOP Level 1	Number of Cases	Max Possible Score	GPT4		Expert 1		Expert 2		Student 1		Student 2		GP 1		GP 2		Mean Human Evaluator Score
1 Dentoalveolar-related pain	6	18	14	78%	15	83%	18	100%	14	78%	12	67%	12	67%	13	72%	78%
2 Myofascial orofacial pain	3	9	3	33%	4	44%	3	33%	3	33%	4	44%	5	56%	4	44%	43%
3 Temporomandibular joint pain	1	3	1	33%	2	67%	2	67%	1	33%	3	100%	3	100%	1	33%	67%
4 Pain related to lesions or disease of the cranial nerves	7	21	10	48%	13	62%	13	62%	12	57%	8	38%	5	24%	13	62%	51%
5 Orofacial pain resembling primary headaches	4	12	6	50%	10	83%	3	25%	3	25%	2	17%	5	42%	4	33%	38%
6 Idiopathic orofacial pain	3	9	2	22%	3	33%	5	56%	7	78%	4	44%	2	22%	2	22%	43%
Total	24	72	36	50%	47	65%	44	61%	40	56%	33	46%	32	44%	37	51%	54%

**Table 8:** GPT4 diagnostic performance for trigeminal neuralgia confirmed cases.

	Trigeminal Neuralgia *n* = 16
Wrong diagnosis	2 (13%)
Correct diagnosis level 1	0 (0%)
Correct diagnosis level 2	1 (6%)
Correct diagnosis level 3	13 (81%)
Total GPT4 diagnostic performance score (0–48)	41 (85%)
Total GPT4 differential quality score (0–80)	74 (93%)

## Data Availability

The data supporting the conclusions of this article will be made available by the authors on request. The dataset generated and analyzed during this study is not publicly available due to potential future commercialization and patenting considerations.

## Supplementary Materials

The following supporting information can be downloaded at https://www.mdpi.com/article/10.3390/diagnostics14242835/s1, Table S1: Prompt design.
