# Supplementary material for: Development and Evaluation of a GPT4-Based Orofacial Pain Clinical Decision Support System
Source: Diagnostics (Basel). 2024 Dec 17;14(24):2835. doi: 10.3390/diagnostics14242835 (PMC11674870; doi:10.3390/diagnostics14242835)
Supplement: Supplementary file 1 [file diagnostics-14-02835-s001.zip › diagnostics-3320646-supplementary.pdf]

GPT4 Prompt

I am running an experiment on complex orofacial and headache pain cases to see how your diagnoses compare with those of human experts. I am going to give you several case descriptions. You are not trying to treat any patients and you must try to give a single definitive diagnosis (though rarely there may be more than one), and it is a diagnosis that is known today to exist in humans. Use the diagnoses and criteria set forward in the "International Classification of Orofacial Pain" or the "International Classification of Headache disorders". Don’t use any other diagnoses or terminology.

You will also notice some scores in each case description. You can find explanations about these in the table below.

Name	Description	Scoring
**FiRST**	Fibromyalgia Rapid Screening Tool (FiRST)	≥5 = likely for fibromyalgia
**HIT-6**	Headache impact test	50-55: some impact on DAL<br>56–59: substantial impact<br>≥60: severe impact
**PCS**	Pain catastrophizing scale	Range from 0-52<br>Higher score = more catastrophizing
**EQ5D**	Euro Quality of Life scores	Subdomains range 1-5<br>1: no impact - 5: extreme impact<br><br>EQ5D VAS range 0-100<br><br>0: worst imaginable QoL - 100: best imaginable QoL
**PHQ9**	Patient health questionnaire assessing depression	0-4: none<br>5-9: mild<br>10-14: moderate<br>15-19: moderately severe<br>20-27: severe

**GAD7**	General anxiety disorder	0-4: no to low risk<br>5-9: mild<br>10-14: moderate<br>15+: severe
**ISI**	Insomnia severity index	0-7: no insomnia<br>8-14: subthreshold insomnia<br>15-21: moderate insomnia<br>22-28: severe insomnia
**Function**	These scales are based on the jaw function limitation scale	Range 0-10<br>0: no impact<br>10: activity completely avoided
**SSS-8**	Somatic symptom scale	0-3: No to minimal<br>4-7: Low<br>8-11: Medium<br>12-15: High<br>16-32: Very high

After you read the case, I want you to give two pieces of information.

The first piece of information is your most likely diagnosis/diagnoses. You need to be as specific as possible -- the goal is to get the correct answer, not a broad category of answers. You do not need to explain your reasoning, just give the diagnosis/diagnoses.

The second piece of information is to give a robust differential diagnosis, ranked by their probability so that the most likely diagnosis is at the top, and the least likely is at the bottom. There is no limit to the number of diagnoses on your differential. You can give as many diagnoses as you think are reasonable. You do not need to explain your reasoning, just list the diagnoses. Again, the goal is to be as specific as possible with each of the diagnoses.

Are you ready for the case?
